# Perceptions, attitudes, practices, and factors associated with COVID-19 vaccination among travelers in the Democratic Republic of the Congo

**DOI:** 10.1186/s40794-024-00240-1

**Published:** 2025-04-15

**Authors:** Harry César Kayembe, Germain Kapour, Papy Ansobi, Aymen Jarboui, Alexis Kalimba Bantu, Glodi Elumbu, Nicodème Nkutu, Eric Mbuyi, Abraham Moyo, Vincent Mbatu, Archilène Nto, Marc Ngondu, Benito Muhindo, Serge Lukunku, Orman Mboyolo, Traoré Ibrahima Sory, Didier Bompangue

**Affiliations:** 1 One Health Institute for Africa, University of Kinshasa, B.P.: 127, Kinshasa, XI Democratic Republic of the Congo; 2 The International Organization for Migration, Kinshasa, Democratic Republic of the Congo

**Keywords:** COVID-19, Vaccination status, Perceptions, Attitudes, Practices, Travelers

## Abstract

**Background:**

Vaccination against COVID-19 has been the primary public health measure implemented to limit the spread of the disease. However, there is still considerable scope for improvement in vaccine coverage, particularly in sub-Saharan African countries. The factors influencing the acceptance or reluctance of the COVID-19 vaccine have been widely studied, but there is a gap in the literature with regard to dynamic populations, particularly travelers, who are one of the priority target groups for vaccination. This study assessed the perceptions, attitudes and practices regarding the COVID-19 vaccine, and explored factors associated with vaccination status among travelers.

**Methods:**

A cross-sectional survey was conducted at several points of entry (PoEs) selected for six survey sites (N’djili airport, Ngobila beach, Lufu, Boma, Moanda, and Kananga), located in three provinces of the Democratic Republic of the Congo (Kinshasa, Kongo Central and Kasaï Central), from February 20 to March 05, 2023. The data were summarized and logistic regression models were performed to assess factors associated with vaccination status.

**Results:**

A total of 2742 travelers were included in this survey. Of these, 54% had received at least one dose of COVID-19 vaccine. Multivariable logistic regression analyses revealed that that several factors were significantly associated with vaccination status. These included age (under 60 years), marital status (single), occupation (other than healthcare worker), mode of travel (other than airplane), and poor perceptions of the vaccine. The most frequently cited reasons for vaccination among respondents who had received the vaccine were the prevention of COVID-19 infection and the ease of travel. In contrast, unvaccinated participants expressed greater concern about the safety and effectiveness of the vaccine, as well as vaccine-related side effects. Furthermore, travel disruption and inappropriate vaccination sites have been identified as significant obstacles to the acceptance of vaccination at the PoEs.

**Conclusions:**

It is essential that awareness initiatives address concerns and misconceptions about vaccine safety and effectiveness. The influence of social media platforms may be harnessed for the dissemination of accurate information from the most trusted information sources, including healthcare professionals, to the target population. In addition, accompanying measures should be considered to facilitate vaccination compliance at different PoEs.

**Supplementary Information:**

The online version contains supplementary material available at 10.1186/s40794-024-00240-1.

## Introduction

As of March 2020, the Democratic Republic of the Congo (DRC) has been facing a pandemic caused by the Coronavirus disease 2019 (COVID-19). In February 2023, the DRC reported more than 95,000 cases and more than 1,400 deaths [1]. However, this epidemiological situation does not reflect the true burden of disease, given the low testing capacity, high test positivity rates during the multiple epidemic waves, and estimates of excess mortality [2–4]. Several control measures have been taken by the government, through the Ministry of Public Health (MoPH), including the introduction of vaccination from April 2021, to deal with the COVID-19 pandemic. By February 2023, over 12,2 million people (10.2% of the total population) received the first dose of COVID-19 vaccine, and 9.7 million people (8.1% of the total population) were fully immunized, including migrants and forcibly displaced people [5].

To boost vaccination coverage, the MoPH opted to revise its national COVID-19 vaccination strategy [6]. Implemented in October 2021, this strategy included creating COVID-19 vaccination sites at the country’s main points of entry (PoE) with a view to achieving the objective of vaccinating 80% of all travelers [6]. To this end, since September 2022, substantial efforts have been made to support the DRC’s MoPH, in particular the Expanded Programme on Immunisation and the National Border Hygiene Programme, by implementing a project to facilitate access to COVID-19 vaccines for international travellers, border users and front-line workers, as well as for cross-border communities. Internally displaced persons and refugees living in formal or informal camps or in host communities were also targeted [5].

Several studies conducted in sub-Saharan Africa show that reluctance to vaccine uptake can be considerable, with hesitancy rates exceeding 90% [7]. Unwavering community support for preventive measures is essential to control the spread of a disease such as COVID-19. However, community adherence depends on a number of factors, including people’s perceptions, attitudes and practices regarding the disease [8, 9]. In addition, the main barriers to vaccine uptake are related to safety and side effects, socio-cultural beliefs and misinformation [7, 10, 11].

Therefore, the main objective of this study is to assess perceptions, attitudes and practices towards the COVID-19 vaccine, and to determine factors associated with vaccination status among priority target groups for COVID-19 vaccination, particularly travelers. To date, there is considerable evidence of acceptance or reluctance to use the COVID-19 vaccine in the general population, health care and school settings [7], but limited studies have focused on dynamic populations as travelers. As evidenced by the literature, the role of travelers in the transmission of infectious diseases is of significant concern. The introduction of a pathogen in a new area by travelers is a key factor in the spread of infectious diseases, including COVID-19 [12–14].

## Methods

### Study design, participants and sampling procedure

**Fig. 1:**
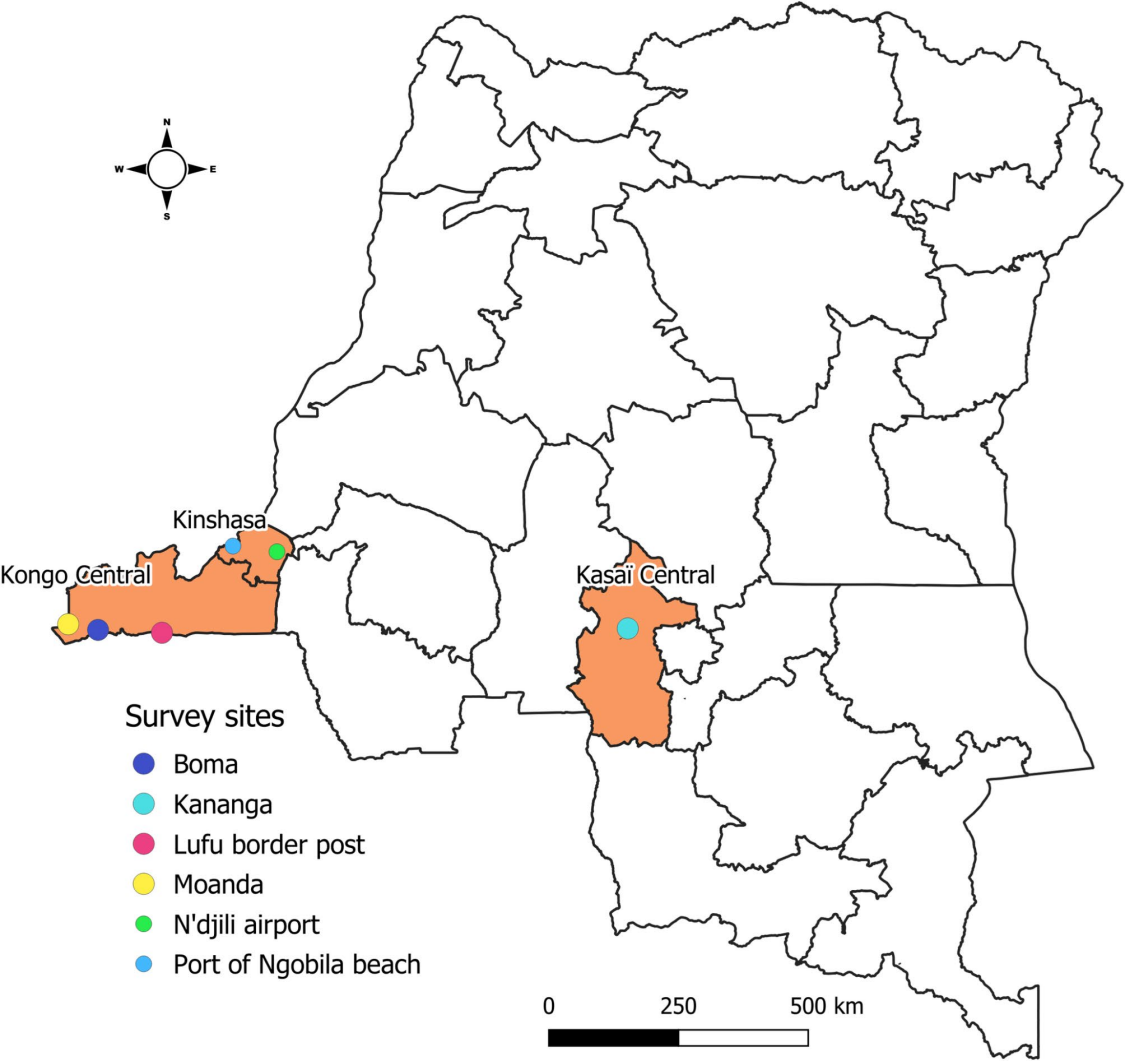
Map of provinces and survey sites selected for this study

A population-based cross-sectional study was conducted among travelers aged 18 years or older who agreed to participate in the survey. The study covered the period from February 20 to March 05, 2023 in three DRC provinces: Kinshasa, Kongo Central and Kasaï Central. For each province, survey sites were selected using convenience sampling based on their significant population flows. The survey sites selected for the country’s densely populated capital, Kinshasa, corresponded to the two main international Points of entry (PoEs): N’djili international airport and port of Ngobila beach (Fig. 1). For Kongo Central province, three survey sites were selected (Fig. 1): the Lufu border post, with two PoEs, a major commercial hub between Kinshasa and Luanda (capital of the Republic of Angola); the city of Boma, with five PoEs, including two ports and three main road stations as transit zones for travelers and goods to and from the border areas of Angola and other neighboring or distant areas and cities of the DRC (Moanda, Tshela, Matadi, Kinshasa, …); Moanda locality, with three PoEs, including a main road station and two border posts as transit zones for travelers and goods to and from the border areas in the north of Angola and other parts of the DRC. For Kasaï Central province, the survey sites selected was the city of Kananga (Fig. 1), with five PoEs, including two main road stations, one port, the railway station, and the international airport.

At the individual level, random sampling was used to select participants. The sample size was calculated using the following formula:

$$\:\text{N}=\frac{{Z}^{2}\:\text{x}\:\text{p}\:(1-\text{p})}{{d}^{2}}$$

Here, N is the sample size, Z is the standard normal variate at 5% significant level (1.96), p represents proportion of travelers who are vaccinated against COVID-19 (50%) as no prior studies reported vaccine coverage among travelers at the time of the study design, d equals tolerable error of margin (0.05). Accounting for a nonresponse rate of 10%, the sample size calculated was 423 participants for each survey site. Finally, we rounded up to 450 individuals for each survey site as a minimum size to increase the power of the statistical test. Therefore, a total sample of 2700 participants were expected for this survey.

### Data collection

Respondents’ data were collected anonymously using a structured questionnaire in electronic form programmed in KoboCollect (https://www.kobotoolbox.org/). The questionnaire was developed in accordance with the findings of several other studies [8, 15–19] and was administered by investigators during a face-to-face interview with respondents. These investigators were pre-recruited and trained to ensure the quality of the use of questionnaire. A preliminary survey of 30 travelers was carried out at a site with similar conditions to those selected to assess the reliability and validity of the tools provided for the formal survey. To this end, the respondents’ reactions to the questionnaire and other aspects such as the length of an interview, the level of understanding of the questions and the difficulties related to informed consent were considered. Finally, the questionnaire included informed consent, sociodemographic characteristics, self-reported information on COVID-19 vaccination, perceptions, attitudes, and practices (Additional file 1).

Sociodemographic characteristics included age, gender, education status, marital status, occupation, means of transport used, and travel reasons [8, 15, 16]. Self-reported information on COVID-19 vaccination focused on vaccination status: (i) if vaccinated: type of vaccine received, number of doses received, episode of COVID-19 infection despite vaccination, and reasons for acceptance of vaccine; (ii) if not vaccinated: reasons for not being vaccinated. The perception section contained eight items related to vaccine protection, vaccine side effects, and misconceptions about the vaccine [17–19]. Three possible responses were suggested: “No”, “Don’t know” and “Yes”, which were coded “0”, “1” and “2”, respectively. The total score calculated by summing the raw scores of all items (ranging from 0 to 16) was used to characterize perceptions as poor (below the mean value) or good (above or equal to the mean value). The attitude section included four items related to COVID-19 concerns, government vaccine approval, vaccine safety and effectiveness, and willingness to be vaccinated [17, 19]. Responses to the items were structured on a three-point Likert scale: “Disagree”, “Undecided” and “Agree”, which were coded “0”, “1” and “2”, respectively. The total score (ranging from 0 to 8) less than the mean value was indicated as negative attitudes, and the total score greater than or equal to the mean value was indicated as positive attitudes. The practices section contained five items on public protection practices [15, 19], with three possible responses (0 = No, 1 = Don’t know, and 2 = Yes). The total score (ranging from 0 to 10) less than the mean value was interpreted as poor practices, and that greater than or equal to the mean value was interpreted as good practices.

### Operational definition

For the purposes of this study, the term “traveler” is defined as any individual who relocates between different geographic locations for any reason and for any length of time [20].

### Statistical analysis

The data collected was imported into Microsoft Excel 2019 files for cleaning, editing, sorting and coding, before being statistically analyzed using R version 4.2.0. Categorical variables were presented as frequencies and percentages, and quantitative variable (age) as median with interquartile range (IQR). Chi-square test and Wilcoxon signed-rank test were used to compare differences between the vaccinated and unvaccinated groups in terms of sociodemographic characteristics and levels of perceptions, attitudes and practices. Binary logistic regression models were used to explore factors associated with COVID-19 vaccination status using crude odds ratios (COR) with their 95% confidence intervals (95% CI). All independent variables with *p*-value < 0.20 in the univariate logistic regression were included in the multivariable logistic regression model. Adjusted odds ratios (AOR) with their 95% CI and *p*-values < 0.05 were employed to ascertain the significant factors and strength of association. For the final multivariable regression model, multicollinearity between the independent variables was evaluated using the variance inflation factor (VIF), with a value of less than 5. Additionally, the model fitting effect was assessed using the Hosmer–Lemeshow goodness-of-fit test, with a result greater than 0.05.

## Results

A total of 2742 travelers agreed to participate in this survey, resulting in an effective response rate of 96%.

### Sociodemographic characteristics of participants

Table 1 summarizes the socio-demographic characteristics of the participants. The median age was 36 (28–46) years. The majority of respondents were 30–44 years old (42.8%), male (63.1%), married (54.4%), and Protestant (46.4%). 39.8% had completed formal secondary education. More than half (59.5%) traveled by vehicle.

**Table 1:** Sociodemographic characteristics of participants

Variables	Total (*n* = 2742)
Age (years), median (IQR)	36 (28–46)
Age group, *n* (%)	
18 to 29 years	806 (29.4)
30 to 44 years	1173 (42.8)
45 to 59 years	594 (21.7)
≥ 60 years	169 (6.2)
Gender, *n* (%)	
Female	1012 (36.9)
Male	1730 (63.1)
Education, *n* (%)	
None	55 (2.0)
Primary	754 (27.5)
Secondary	1091 (39.8)
Tertiary	842 (30.7)
Marital status, *n* (%)	
Married	1492 (54.4)
Single	1150 (41.9)
Other^a^	100 (3.6)
Religion, *n* (%)	
Catholicism	730 (26.6)
Protestant	1272 (46.4)
Muslim	96 (3.5)
Other^b^	644 (23.5)
Occupation, *n* (%)	
Healthcare worker	122 (4.4)
Trader	743 (27.1)
Civil servant	361 (13.2)
Unemployed	754 (27.5)
Other^c^	762 (27.8)
Means of transport, *n* (%)	
Airplane	450 (16.4)
Boat	621 (22.6)
Vehicle	1631 (59.5)
Train	7 (0.3)
Other (walking or bicycling)	33 (1.2)

Other^a^: Divorced/Separated, Widowed; Other^b^: Animist, Without religion; Other^c^: Liberal profession

Work was the most frequently cited reason for travel in Kinshasa, Ndjili airport (31%) and Ngobila beach (28%), while business was the main reason for travel in other survey sites, Boma (36%), Lufu (52%), Moanda (33%) and Kananga (33%) (Additional file 2).

### Perceptions, attitudes, and practices towards the COVID-19 vaccine

Table 2 shows that 61.9% of the participants indicated that the COVID-19 vaccine can protect against re-infection with the disease. 43% of respondents thought that mass vaccination of the population can provide indirect protection for non-vaccinated people, and 38.9% believed that a single dose of COVID-19 vaccine is sufficient to acquire immunity against the disease. 45.4% of individuals responded that vaccinating travelers at PoEs could eliminate COVID-19. A not insignificant proportion of the participants perceived the vaccine to have side effects (54.9%) and to be unsuitable for anyone, regardless of age (42.1%). A total of 45.4% and 42.1% of the participants, respectively, indicated uncertainty regarding the administration of the COVID-19 vaccine to individuals with known allergies or chronic diseases.

**Table 2:** Perceptions of respondents towards COVID-19 vaccine

Perceptions	*n* (%)	95% CI
Vaccine protects against re-infection with COVID-19		
Yes	1697 (61.9)	60.0-63.7
No	411 (15.0)	13.7–16.2
Don’t know	634 (23.1)	21.5–24.7
Mass vaccination of the population provides indirect protection for non-vaccinated people		
Yes	1179 (43.0)	41.1–44.7
No	814 (29.7)	28.0-31.4
Don’t know	749 (27.3)	25.7–28.9
Vaccine can confer total immunity with a single dose		
Yes	1067 (38.9)	37.1–40.8
No	628 (22.9)	21.3–24.5
Don’t know	1047 (38.2)	36.4–40.0
COVID-19 could be eliminated by vaccinating travelers at PoEs		
Yes	1244 (45.4)	43.5–47.2
No	778 (28.4)	26.7–30.1
Don’t know	720 (26.3)	24.5–28.0
Vaccine can be administered to anyone, regardless of age		
Yes	813 (29.6)	27.9–31.3
No	1154 (42.1)	40.3–44.0
Don’t know	775 (28.3)	26.6–30.1
Vaccine has side effects		
Yes	1505 (54.9)	52.9–56.8
No	608 (22.2)	20.6–23.7
Don’t know	629 (22.9)	21.3–24.5
Vaccine can be administered to people with known allergies		
Yes	764 (27.9)	26.3–29.4
No	734 (26.8)	25.1–28.5
Don’t know	1244 (45.4)	43.5–47.2
Vaccine can be administered to people with chronic diseases (hypertension, diabetes, …)		
Yes	906 (33.0)	31.4–34.8
No	682 (24.9)	23.3–26.4
Don’t know	1154 (42.1)	40.2–43.9

95% CI: 95% confidence intervals

Table 3 demonstrates that 60.5% of the participants admitted to be concerned about COVID-19. Approximately 69% of respondents agreed with government-approved vaccines, while 54% agreed with vaccine safety and effectiveness. 63.2% of travelers agreed to vaccination (revaccination) at the PoE.

**Table 3:** Attitudes of respondents towards COVID-19 vaccine

Attitudes	*n* (%)	95% CI
Concerned about COVID-19		
Agree	1659 (60.5)	58.6–62.3
Disagree	940 (34.3)	32.6–36.2
Undecided	143 (5.2)	4.3–6.1
Confidence in government-approved vaccines		
Agree	1873 (68.3)	66.6–70.1
Disagree	685 (25.0)	23.4–26.6
Undecided	184 (6.7)	5.8–7.6
Confidence in vaccine safety and effectiveness		
Agree	1481 (54.0)	52.1–56.0
Disagree	1036 (37.8)	36.0-39.6
Undecided	225 (8.2)	7.2–9.2
Willing to get vaccinated (revaccinated) at the PoE		
Agree	1733 (63.2)	61.2–65.0
Disagree	930 (33.9)	32.2–35.7
Undecided	79 (2.9)	2.2–3.6

95% CI: 95% confidence intervals

The majority of participants reported not washing their hands frequently (58.5%), not wearing masks (88.5%) and not keeping social distance (94.7%) in closed public places, not covering their mouths and noses when coughing or sneezing (75.5%), and not being tested for COVID-19 in the presence of signs suggestive of the disease (83%) (Table 4).

**Table 4:** Practices of respondents towards COVID-19

Practices	*n* (%)	95% CI
Frequent hand washing		
Yes	1119 (40.8)	38.9–42.7
No	1603 (58.5)	56.6–60.4
Don’t know	20 (0.7)	0.4–1.1
Wearing masks in enclosed public places		
Yes	315 (11.5)	10.4–12.8
No	2427 (88.5)	87.2–89.6
Social distancing in enclosed public places		
Yes	144 (5.3)	4.4–6.1
No	2598 (94.7)	93.9–95.6
Covering mouth and nose when coughing or sneezing		
Yes	657 (24.0)	22.4–25.6
No	2071 (75.5)	73.9–77.1
Don’t know	14 (0.5)	0.3–0.8
Testing for COVID-19 when signs suggestive of the disease		
Yes	467 (17.0)	15.5–18.6
No	2275 (83.0)	81.4–84.5

95% CI: 95% confidence intervals

### Comparison of sociodemographic characteristics, perceptions, attitudes, and practices according to vaccination status

Table 5 displays comparisons between vaccinated and unvaccinated groups according to sociodemographic characteristics, perceptions, attitudes, and practices. Overall, 1474 (54%) of the 2742 participants included in the survey had received at least one dose of a COVID-19 vaccine. The median age in the vaccinated group, 38 (30–49) years, was significantly higher than in the non-vaccinated group, 34 (27–43) years. The proportion of vaccinated individuals was higher among those aged 30 and older, with a secondary or high education level, and who were married. Unvaccinated participants were more likely to be unemployed, and to adhere to other religious beliefs, compared with vaccinated participants. They were also more likely to travel by vehicle and bicycle or on foot. In addition, respondents who had been vaccinated were more likely to have good perceptions, positive attitudes, and good practices towards COVID-19 vaccination compared with those who had not been vaccinated.

**Table 5:** Comparison of sociodemographic characteristics, perceptions, attitudes, and practices of participants according to vaccination status

Variables	Unvaccinated group (*n* = 1268)	Vaccinated group (*n* = 1474)	*p*-value
Age, median (IQR)	34 (27–43)	38 (30–49)	**< 0.001**
Age group, *n* (%)			**< 0.001**
18 to 29 years	439 (54.5)	367 (45.5)	
30 to 44 years	560 (47.7)	613 (52.3)	
45 to 59 years	233 (39.2)	361 (60.8)	
≥ 60 years	36 (21.3)	133 (78.7)	
Gender, *n* (%)			0.074
Female	445 (44.0)	567 (56.0)	
Male	823 (47.6)	907 (52.4)	
Education, *n* (%)			**< 0.001**
None	29 (52.7)	26 (47.3)	
Primary	441 (58.5)	313 (41.5)	
Secondary	534 (48.9)	557 (51.1)	
Tertiary	264 (31.4)	578 (68.6)	
Marital status, *n* (%)			**< 0.001**
Married	603 (40.4)	889 (59.6)	
Single	623 (54.2)	527 (45.8)	
Other^a^	42 (42.0)	58 (58.0)	
Religion, *n* (%)			**< 0.001**
Catholicism	288 (39.5)	442 (60.5)	
Protestant	605 (47.6)	667 (52.4)	
Muslim	44 (45.8)	52 (54.2)	
Other^b^	331 (51.4)	313 (48.6)	
Occupation, *n* (%)			**< 0.001**
Healthcare worker	19 (15.6)	103 (84.4)	
Trader	340 (45.8)	403 (54.2)	
Civil servant	136 (37.7)	225 (62.3)	
Unemployed	410 (54.4)	344 (45.6)	
Other^c^	363 (47.6)	399 (52.4)	
Means of transport, *n* (%)			**< 0.001**
Airplane	48 (10.7)	402 (89.3)	
Boat	303 (48.8)	318 (51.2)	
Vehicle	888 (54.4)	743 (45.6)	
Train	2 (28.6)	5 (71.4)	
Other (walking or bicycling)	27 (81.8)	6 (18.2)	
Perceptions, *n* (%)			**< 0.001**
Good	537 (35.4)	981 (64.6)	
Poor	731 (59.7)	493 (40.3)	
Attitudes, *n* (%)			**< 0.001**
Positive	571 (34.2)	1097 (65.8)	
Negative	697 (64.9)	377 (35.1)	
Practices, *n* (%)			**< 0.001**
Good	620 (36.1)	1096 (63.9)	
Poor	648 (63.2)	378 (36.8)	

Other^a^: Divorced/Separated, Widowed; Other^b^: Animist, Without religion; Other^c^: Liberal profession

Among vaccinated travelers, 42.3% had received a Johnson & Johnson vaccine, followed by Pfizer (14.8%), Moderna (12.9%) and AstraZeneca (10.4%). 17.4% could not recall which vaccine they had received (Additional file 3). With the exception of the Johnson & Johnson vaccine (93.7% single dose), less than 70% of vaccinated participants had received two doses of each vaccine. Six out of ten vaccinated respondents who could not recall the type of vaccine had received a single dose (Additional file 4). Furthermore, the main reasons cited by travelers for vaccinating against COVID-19 were disease prevention (49.3%), ease of travel (19.7%), and disease awareness (15.0%) (Additional file 5). Healthcare workers (HCWs) were more likely to be vaccinated because of disease prevention (71.3%), awareness (23.0%), and a history of COVID-19 infection (4.9%), while traders cited ease of travel (18.4%) and fear of disease (4.0%) (Additional file 6). In addition, 10% of participants admitted to having contracted COVID-19 despite vaccination (Additional file 7).

Among individuals who have not received the vaccination, the most common reason for not doing so was fear of side effects (37.1%). A total of 29.3% of unvaccinated individuals indicated that they believed the vaccine to be unsafe and ineffective, while 16.2% stated that they did not believe the disease to be real (Additional file 8). Furthermore, 57% of respondents did not agree to get vaccinated at the PoE surveyed because of the risk of travel disruption, while 38% said the site was unsuitable for vaccination (Additional file 9).

### Factors associated with COVID-19 vaccination status

In univariate logistic regression analysis, it was found that age, education, marital status, religion, occupation, means of transport, perceptions, attitudes, and practices were statistically significantly associated with vaccination status. In the final multivariable logistic regression analysis (Table 6), respondents in the age groups of 18 to 29 years (AOR: 1.85, 95% CI: 1.16-3.00), 30 to 44 years (AOR: 1.90, 95% CI: 1.23–2.99), and 45 to 59 years (AOR: 1.78, 95% CI: 1.14–2.83) were less likely to get the COVID-19 vaccine, compared to those aged 60 years or older. Single individuals were more likely to be unvaccinated than married individuals (AOR: 1.78, 95% CI: 1.45–2.18). Civil servants (AOR: 2.61, 95% CI: 1.47–4.81), traders (AOR: 2.20, 95% CI: 1.27-4.00), and other professional categories (AOR: 2.60, 95% CI: 1.50–4.70), as well as the unemployed (AOR: 2.55, 95% CI: 1.45–4.65) were found to be less likely vaccinated than healthcare workers. Respondents who travelled by foot or bicycle (AOR: 26.62, 95% CI: 10.59–77.21), vehicle (AOR: 11.55, 95% CI: 8.19–16.60), and boat (AOR: 8.06, 95% CI: 5.67–11.65) were more likely to be in unvaccinated group than those who flew. People with poor perceptions towards COVID-19 vaccine were more hesitant to get vaccinated than those with good perceptions (AOR: 3.42, 95% CI: 2.87–4.09).

**Table 6:** Logistic regression analysis of factors affecting COVID-19 vaccination status among travelers

Variables	COR (95% CI)	*p*-value	AOR (95% CI)	*p*-value
Age group				
18 to 29 years	4.42 (3.01–6.63)	**< 0.001**	1.85 (1.16-3.00)	**0.011**
30 to 44 years	3.38 (2.32–5.03)	**< 0.001**	1.90 (1.23–2.99)	**0.005**
45 to 59 years	2.38 (1.61–3.61)	**< 0.001**	1.78 (1.14–2.83)	**0.013**
≥ 60 years	Reference		Reference	
Gender				
Female	Reference			
Male	1.16 (0.99–1.35)	0.068		
Education				
None	Reference		Reference	
Primary	1.26 (0.73–2.19)	0.404	1.30 (0.70–2.40)	0.404
Secondary	0.86 (0.50–1.48)	0.584	0.98 (0.53–1.80)	0.945
Tertiary	0.41 (0.24–0.71)	**0.001**	0.82 (0.43–1.54)	0.538
Marital status				
Married	Reference		Reference	
Single	1.74 (1.49–2.04)	**< 0.001**	1.78 (1.45–2.18)	**< 0.001**
Other^a^	1.07 (0.70–1.60)	0.755	1.14 (0.71–1.83)	0.597
Religion				
Catholicism	0.62 (0.50–0.76)	**< 0.001**		
Protestant	0.86 (0.71–1.04)	0.113		
Muslim	0.80 (0.52–1.23)	0.310		
Other^b^	Reference			
Occupation				
Healthcare worker	Reference		Reference	
Trader	4.52 (2.81–7.84)	**< 0.001**	2.20 (1.27-4.00)	**0.007**
Civil servant	3.28 (1.96–5.73)	**< 0.001**	2.61 (1.47–4.81)	**0.002**
Unemployed	6.46 (3.97–11.07)	**< 0.001**	2.55 (1.45–4.65)	**0.002**
Other^c^	4.93 (3.03–8.44)	**< 0.001**	2.60 (1.50–4.70)	**< 0.001**
Means of transport				
Airplane	Reference		Reference	
Boat	7.98 (5.74–11.30)	**< 0.001**	8.06 (5.67–11.65)	**< 0.001**
Vehicle	10.01 (7.38–13.87)	**< 0.001**	11.55 (8.19–16.60)	**< 0.001**
Train	3.35 (0.47–16.01)	0.155	4.46 (0.60-23.06)	0.092
Other (walking or bicycling)	37.69 (15.77–105.20)	**< 0.001**	26.62 (10.59–77.21)	**< 0.001**
Perceptions				
Good	Reference		Reference	
Poor	2.71 (2.32–3.17)	**< 0.001**	3.42 (2.87–4.09)	**< 0.001**
Attitudes				
Positive	Reference			
Negative	3.55 (3.03–4.18)	**< 0.001**		
Practices				
Good	Reference			
Poor	3.03 (2.58–3.56)	**< 0.001**		

95% CI: 95% confidence interval; AOR: Adjusted odd ratio; COR: Crude odd ratio; Other^a^: Divorced/Separated, Widowed; Other^b^: Animist, Without religion; Other^c^: Liberal profession

## Discussion

This population-based cross-sectional study, conducted in three DRC provinces (Kinshasa, Kongo Central, and Kasaï Central), aimed to assess perceptions, attitudes and practices towards the COVID-19 vaccine, and to explore factors associated with vaccination status among travelers, one of the priority target groups for COVID-19 vaccination. The findings revealed that 54% of the respondents had received at least one dose of a COVID-19 vaccine. The variables of age, marital status, occupation, means of transport, and perceptions were found to exert a significant influence on vaccination status.

The vaccine coverage found in this study was much higher than that reported in the general population at the time of the study design, with 10.2% having received at least one dose of a COVID-19 vaccine [5]. A recent study carried out in the city of Kinshasa reported 15% coverage [21]. It is notable that travelers represent a relatively minor proportion of the general population, particularly in the context of air travel, where socio-economic conditions may afford individuals the opportunity to fly. Despite this, the population in question demonstrated a proclivity to accept the vaccination in response to the country-specific requirements that were implemented to facilitate national and international travel [22–24]. This also corroborates the findings of our study, which indicated that the second most frequently cited reason for accepting the COVID-19 vaccine was ease of travel. It has been demonstrated that the relationship between attitudes toward the COVID-19 vaccine and intentions to receive the COVID-19 vaccination is significantly influenced by the desire to travel [25].

The primary rationale for vaccine acceptance among those who had received the vaccine was the prevention of COVID-19 infection. This was the most frequently cited reason by HCWs. These individuals have been identified as a priority target for early vaccination against SARS-CoV-2 infection due to their higher risk of exposure to and transmission of the disease than other occupational categories [26, 27]. Nevertheless, the prevalence of vaccine hesitancy among HCWs across the African continent remains relatively high [7]. Furthermore, a recent study conducted in seven DRC provinces, including two provinces selected for inclusion in our survey, reported an acceptance rate of 46.3% among HCWs [2].

This study revealed that individuals under the age of 60 and single individuals were less likely to be vaccinated than those aged 60 and over, and married individuals, respectively. It is presumed that older adults were more aware of their elevated susceptibility to severe outcomes of SARS-CoV-2 and the efficacy of the vaccine in mitigating them [28]. However, the elderly are also susceptible to vaccine-related adverse effects and a diminished immune response to vaccination [29, 30]. With regard to marital status, it is plausible that unmarried individuals, who are typically younger, are more vulnerable to misinformation and rumors about the COVID-19 vaccine due to their greater access to information, with the Internet, particularly social and digital media platforms, serving as the primary source [31–33].

Our findings indicate that individuals who utilized alternative modes of transportation, such as walking, bicycling, vehicles, and boats, were more likely to be unvaccinated compared to those who flew. These findings highlight that increased efforts to enhance control measures, including the administration of vaccines against COVID-19, have been particularly relevant for air travelers, while other traveler subgroups have received comparatively less attention. In the context under consideration, it has been demonstrated that the primary focus of the verification process of COVID-19 vaccination certificates for travel was at the airport. Travelers not fully vaccinated were obliged to undergo a test for COVID-19 upon arrival in the country or prior to undertaking any travel within the country [21, 22]. Furthermore, it would have been beneficial to promote additional measures to enhance compliance with the COVID-19 vaccination program through the implementation of awareness campaigns at primary transportation hubs, main road stations, border posts, ports, railway stations, as well as all other PoEs. However, the disruption of travel and the presence of unsuitable vaccination sites have been identified as significant obstacles to the acceptance of vaccination at PoEs. To address these concerns, it is essential to implement tailored accompanying measures.

The present study demonstrated that travelers with unfavorable perceptions of the COVID-19 vaccine exhibited greater reluctance to be vaccinated than those with favorable perceptions. The primary reasons provided by unvaccinated respondents in the survey for their refusal to be vaccinated were concerns about vaccine safety and effectiveness, as well as concerns about potential adverse events associated with the vaccine. These factors have been identified as potential significant determinants of hesitancy or refusal of the COVID-19 vaccine [7, 34], given that individuals in poorer regions are more likely to encounter and believe rumors and misinformation about COVID-19 vaccination [7, 10, 11, 35]. Therefore, our findings are applicable to low- and middle-income countries in other regions, where a higher prevalence of misinformation has also been documented [35]. Furthermore, in order to overcome vaccine misinformation and misconceptions, it is crucial to leverage the influence of social media platforms for the dissemination of accurate information from the most trusted information sources, including healthcare professionals, to the target population, given the prominence of digital media in the current era.

It should be acknowledged that this study is not without certain limitations. The provinces included in this study were selected on the basis of time and resource constraints. Consequently, we endeavored to incorporate a diverse array of PoEs within each survey site. To ensure the generalizability of our findings at the national level, it is necessary to include a broader geographic representation. Secondly, the self-reported vaccination status of respondents may have been influenced by social desirability and recall biases. Such reporting practices could also result in an overestimation or underestimation of the proportion of individuals who have received the vaccination. Thirdly, the cross-sectional design of our study did not allow us to ascertain a causal relationship between the participants’ socio-demographic characteristics, perceptions, attitudes and practices and their COVID-19 vaccination status. Given the expected variation in estimated coverage and vaccine hesitancy or refusal over time, longitudinal studies are necessary to provide regular updates to related information in line with the evolution of the epidemiological context.

To the best of our knowledge, this study is one of the few to address the factors associated with the COVID-19 vaccination status of travelers, who are broadly representative of all vaccine target groups. Our findings provide a foundation for health policymakers and planners seeking to enhance vaccination rates within the general population, particularly among target groups. In a broader context, the results of this study provide a basis to develop and implement evidence-based initiatives for vaccination programs to control other travel-related infectious diseases.

## Conclusions

COVID-19 vaccination coverage was relatively low among dynamic populations such as travelers, one of the priority target groups for vaccination. The results of our study indicate the necessity for augmented endeavors to devise or integrate tailored awareness initiatives with the objective of enhancing health literacy and fostering public confidence in vaccination. As the primary conduit for disseminating information [36], the influence of social media platforms must be considered when developing accurate vaccination messages to counteract misinformation that is readily accepted by the general public. Additionally, flexibility measures should be incorporated into the establishment of vaccination sites to enhance compliance with vaccination procedures at various PoEs.

## Electronic supplementary material

Below is the link to the electronic supplementary material.

Supplementary Material 1

Supplementary Material 2

Supplementary Material 3

Supplementary Material 4

Supplementary Material 5

Supplementary Material 6

Supplementary Material 7

Supplementary Material 8

Supplementary Material 9

Supplementary Material 10

## Data Availability

All data generated or analyzed during this study are included in this published article.

## Abbreviations

95% CI: 95% Confidence intervals
AOR: Adjusted odds ratios
COR: Crude odds ratios
DRC: Democratic republic of the congo
HCWs: Healthcare workers
IQR: Interquartile range
MoPH: Ministry of public health
PoEs: Points of entry
VIF: Variance inflation factor
