# Supplementary material for: Perceptions, attitudes, practices, and factors associated with COVID-19 vaccination among travelers in the Democratic Republic of the Congo
Source: Trop Dis Travel Med Vaccines. 2025 Apr 15;11:10. doi: 10.1186/s40794-024-00240-1 (PMC11998446; doi:10.1186/s40794-024-00240-1)
Supplement: Supplementary file 1 — Supplementary Material 1 [file 40794_2024_240_MOESM1_ESM.docx]

**Questionnaire d’enquête sur les Perceptions, attitudes et pratiques des voyageurs sur la vaccination contre le COVID-19 en République Démocratique du Congo**

***Survey questionnaire on travellers' perceptions, attitudes and practices regarding vaccination against COVID-19 in the Democratic Republic of the Congo***

**Consentement éclairé**

Monsieur/Madame/Mademoiselle,

Nous réalisons une enquête sur les perceptions, attitudes et pratiques des voyageurs sur la vaccination contre la COVID-19 en RDC. Les résultats qui en découleront permettront de contribuer à l’ajustement des stratégies visant à améliorer la couverture vaccinale contre la COVID-19 dans notre pays. Votre participation, libre et volontaire, sera d’une grande utilité pour atteindre cet objectif. Et aussi, les informations que vous donnerez resteront confidentielles. La durée totale de cet entretien ne pourra dépasser 15 minutes.

Acceptez-vous de répondre à nos questions ?

- Oui
- Non

Si Oui, passer à la question 001. Si non, remercier la personne et clôturer l’entretien.

***Informed consent***

*Dear Sir/Madam/Miss,*

*We are conducting a survey on travellers' perceptions, attitudes and practices regarding vaccination against COVID-19 in the DRC. The results will be used to help adjust strategies aimed at improving vaccination coverage against COVID-19 in our country. Your free and voluntary participation will be of great help in achieving this objective. The information you provide will remain confidential. The interview should last no longer than 15 minutes.*

*Are you willing to answer our questions?*

- *Yes*
- *No*

*If yes, go on to question 001. If No, thank the person and close the interview.*

**I. Caractéristiques sociodémographiques des voyageurs** */* ***Socio-demographic characteristics of travellers***

| **N°** | **Question** | **Réponses / *Answers*** |
| --- | --- | --- |
| 001 | Quel âge avez-vous?  *How old are you ?* |  |
| 002 | Sexe de l’enquêté  *Gender of the respondent* | - Féminin / *Female* - Masculin / *Male* |
| 003 | Quel est votre niveau d’étude ?  *What is your level of education?* | - Aucun / *None* - Primaire / *Primary* - Secondaire / *Secondary* - Supérieur / *Tertiary* |
| 004 | Quel est votre statut matrimonial ?  *What is your marital status?* | - Célibataire / *Single* - Marié(e) / *Married* - Autre (préciser) / *Other (specify)* |
| 005 | Quelle est votre religion ?  *What is your religion?* | - Catholique / *Catholicism* - Protestante / *Protestant* - Islam / *Muslim* - Autre (préciser) / *Other (specify)* |
| 006 | Quelle est votre occupation actuelle ?  *What is your current occupation?* | - Professionnel de santé / *Healthcare worker* - Fonctionnaire / *Civil servant* - Commerçant / *Trader* - Sans emploi formel / *Unemployed* - Autre (préciser) / *Other (specify)* |
| 007 | Quel est le moyen de transport utilisé pour effectuer ce voyage ?  *What means of transport did you use to make this trip?* | - Véhicule / *Vehicle* - Bateau / *Boat* - Avion / *Airplane* - Train / *Train* - Autre (marche ou vélo) / *Other (walking or bicycling)* |
| 008 | Pour quelle raison effectuez-vous ce voyage ?  *Why are you making this trip?* | - Commerce / *Trade* - Travail / *Work* - Visite familiale / *Family visit* - Tourisme / *Tourism* - Soins médicaux / *Medical care* - Etudes / Studies - Autre (préciser) / *Other (specify)* |

**II. Information sur la couverture vaccinale contre la COVID-19 / *Information on vaccination coverage against COVID-19***

| **N°** | **Question** | **Réponses / *Answers*** |
| --- | --- | --- |
| 009 | Avez-vous été vacciné contre la COVID-19 ?  *Have you been vaccinated against COVID-19?* | - Oui / *Yes* - Non / *No*   **Si « non », passer à la question 014 / If "no", go to question 014* |
| 010 | Quel vaccin vous a été administré ?  What vaccine were you given? | - Astra Zeneca - Pfizer - Moderna - Johnson & Johnson - Sinopharm - Je ne sais pas / *I don’t know* |
| 011 | Combien de doses de vaccin aviez-vous reçu ?  *How many doses of vaccine had you received?* | - 1 dose - 2 doses - > 2 doses |
| 012 | Quelles sont les raisons qui vous ont poussé à vous faire vacciner ?  *What are your reasons for getting vaccinated?* | - Prévention contre la maladie / *Disease prevention* - Sensibilisation sur la maladie / *Awareness of the disease* - Par contrainte / *By constraint* - Pour voyager aisément / *Travel with ease* - Antécédent de la COVID-19 / *History of COVID-19 infection* - Autre (préciser) / *Other (specify)* |
| 013 | Avez-vous contracté le COVID-19 malgré la vaccination ?  *Have you contracted COVID-19 despite having been vaccinated?* | - Oui / *Yes* - Non / *No* |
| 014 | Pourquoi êtes-vous réticent à vous faire vacciner ?  *Why are you reluctant to get vaccinated?* | - La maladie n’existe pas / *The disease does not exist* - La maladie a disparu / *The disease has disappeared* - Par peur des effets indésirables / *Fear of side effects* - Confiance en Dieu / *Trust in God* - Le vaccin est dangereux et inefficace / *Vaccine is unsafe and ineffective* - Sites de vaccination non connus / *Vaccination sites unknown* - Je suis en bonne santé / *I am in good health* - Mauvaise attitude du personnel soignant / *Poor attitude of HCWs* |

**III. Perceptions**

| **N°** | **Question** | **Réponses / *Answers*** |
| --- | --- | --- |
| 015 | Le vaccin contre la COVID-19 offre-t-il une protection qui empêche de contracter à nouveau le virus de la COVID-19 ?  *Does the COVID-19 vaccine offer protection against re-infection with the COVID-19 virus?* | - Oui / *Yes* - Non / *No* - Je ne sais pas / *I don’t know* |
| 016 | La vaccination de masse de la population offre-t-elle une protection indirecte à ceux qui n'ont pas été vaccinés ?  *Does mass vaccination of the population offer indirect protection to those who have not been vaccinated?* | - Oui / *Yes* - Non / *No* - Je ne sais pas / *I don’t know* |
| 017 | La prise d'une seule dose du vaccin est-elle suffisante pour acquérir une immunité contre le virus de la COVID-19 ?  *Is a single dose of vaccine sufficient to acquire immunity against COVID-19?* | - Oui / *Yes* - Non / *No* - Je ne sais pas / *I don’t know* |
| 018 | La vaccination des voyageurs aux points d'entrée pourrait-elle permettre d'éliminer le COVID-19 ?  *Could vaccination of travelers at PoEs eliminate COVID-19?* | - Oui / *Yes* - Non / *No* - Je ne sais pas / *I don’t know* |
| 019 | Le vaccin contre la COVID-19 peut-il être administré à n’importe qui, quel que soit son âge ?  *Can the COVID-19 vaccine be administered to anyone, regardless of age?* | - Oui / *Yes* - Non / *No* - Je ne sais pas / *I don’t know* |
| 020 | Le vaccin contre la COVID-19 a-t-il des effets secondaires ?  *Does the COVID-19 vaccine have any side effects?* | - Oui / *Yes* - Non / *No* - Je ne sais pas / *I don’t know* |
| 021 | Les personnes souffrant d'allergies connues peuvent-elles recevoir le vaccin contre la COVID-19 ?  *Can people with known allergies receive the COVID-19 vaccine?* | - Oui / *Yes* - Non / *No* - Je ne sais pas / *I don’t know* |
| 022 | Les personnes souffrant de maladies chroniques peuvent-elles recevoir le vaccin contre la COVID-19 ?  *Can people with chronic diseases receive the COVID-19 vaccine?* | - Oui / *Yes* - Non / *No* - Je ne sais pas / *I don’t know* |

**IV. Attitudes**

| **N°** | **Déclarations / *Statements*** | **Réponses / *Answers*** |
| --- | --- | --- |
| 023 | Vous vous sentez toujours concerné par la pandémie à COVID-19  *You are still concerned about the COVID-19 pandemic* | - D’accord / *Agree* - Pas d’accord / *Disagree* - Indécis / *Undecided* |
| 024 | Vous avez une confiance totale envers les vaccins autorisés par le Ministère de la Santé  *You have complete confidence in the vaccines approved by the governement* | - D’accord / *Agree* - Pas d’accord / *Disagree* - Indécis / *Undecided* |
| 025 | Vous avez une confiance totale dans la sécurité et l'efficacité des vaccins  *You have complete confidence in the safety and efficacy of vaccines*. | - D’accord / *Agree* - Pas d’accord / *Disagree* - Indécis / *Undecided* |
| 026 | Vous pouvez vous faire vacciner à ce point d’entrée sans problème  You can get vaccinated (re-vaccinated) at this PoE without any problem | - D’accord / *Agree* - Pas d’accord / *Disagree* - Indécis / *Undecided* |

**V. Pratiques / *Practices***

| **N°** | **Question** | **Réponses** |
| --- | --- | --- |
| 027 | Vous lavez-vous les mains fréquemment ?  *Do you wash your hands frequently?* | - Oui / *Yes* - Non / *No* - Je ne sais pas / *I don’t know* |
| 028 | Portez-vous le masque dans les lieux publics fermés ?  *Do you wear a mask in enclosed public places?* | - Oui / *Yes* - Non / *No* - Je ne sais pas / *I don’t know* |
| 029 | Pratiquez-vous la distanciation sociale dans les lieux publics fermés ?  *Do you practice social distancing in enclosed public places?* | - Oui / *Yes* - Non / *No* - Je ne sais pas / *I don’t know* |
| 030 | Vous couvrez-vous la bouche et le nez avec le pli du coude quand vous toussez ou éternuez ?  *Do you cover your mouth and nose with the crease of your elbow when you cough or sneeze?* | - Oui / *Yes* - Non / *No* - Je ne sais pas / *I don’t know* |
| 031 | Vous faites-vous tester à la COVID-19 lorsque vous présenter des signes évocateurs de la maladie ?  *Do you get tested for COVID-19 when you show signs suggestive of the disease?* | - Oui / *Yes* - Non / *No* - Je ne sais pas / *I don’t know* |
