# Supplementary figures and images for: Perceptions, attitudes, practices, and factors associated with COVID-19 vaccination among travelers in the Democratic Republic of the Congo
Source: Trop Dis Travel Med Vaccines. 2025 Apr 15;11:10. doi: 10.1186/s40794-024-00240-1 (PMC11998446; doi:10.1186/s40794-024-00240-1)

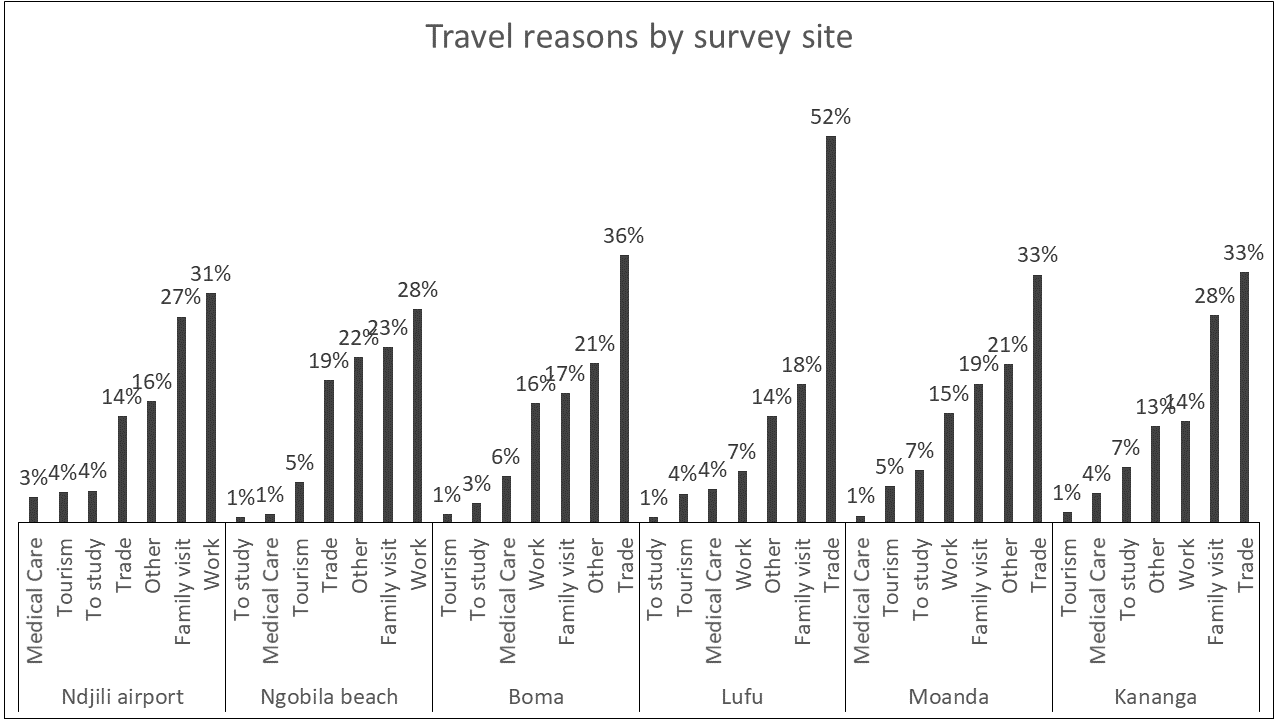

Supplement: Supplementary file 2 — Supplementary Material 2 [file 40794_2024_240_MOESM2_ESM.tif]

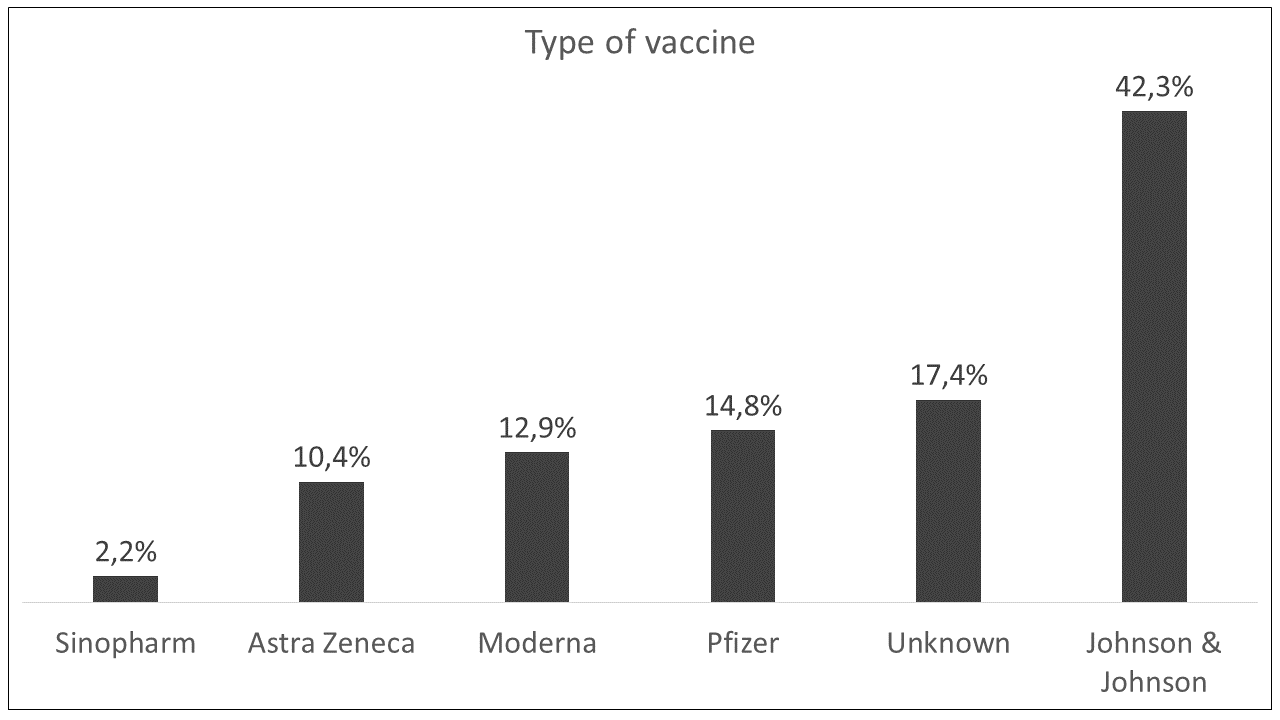

Supplement: Supplementary file 3 — Supplementary Material 3 [file 40794_2024_240_MOESM3_ESM.tif]

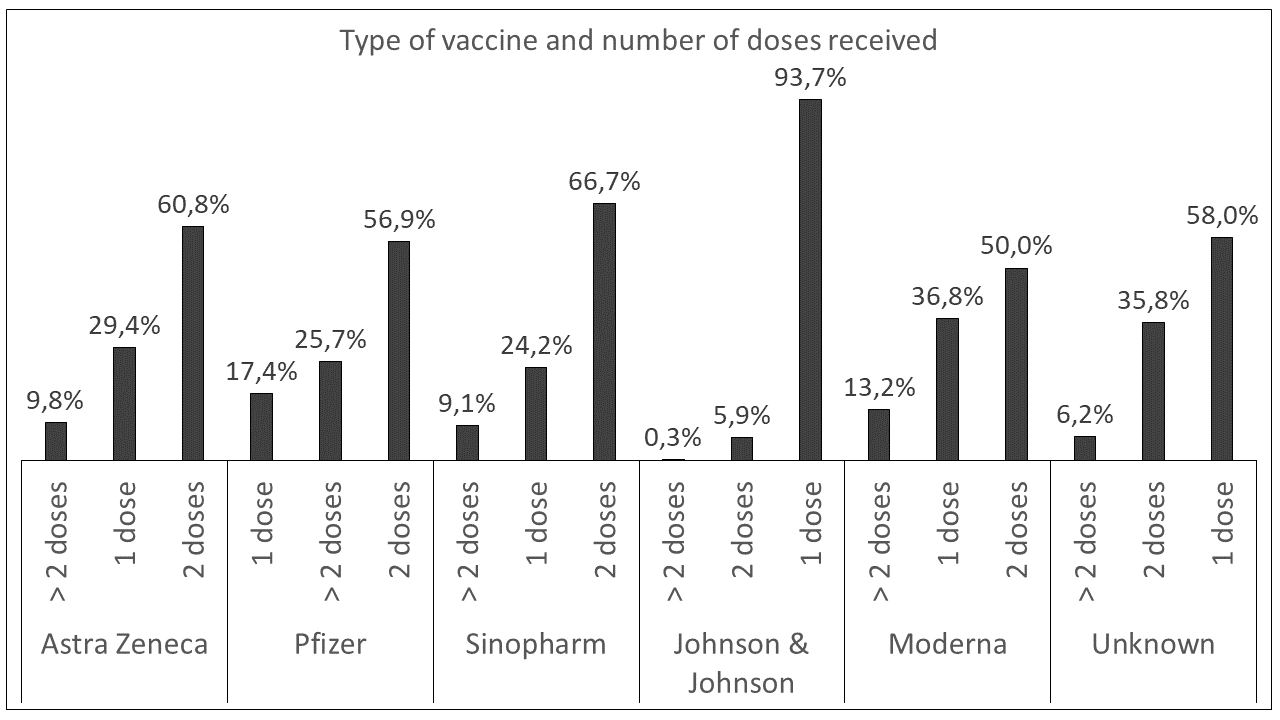

Supplement: Supplementary file 4 — Supplementary Material 4 [file 40794_2024_240_MOESM4_ESM.tif]

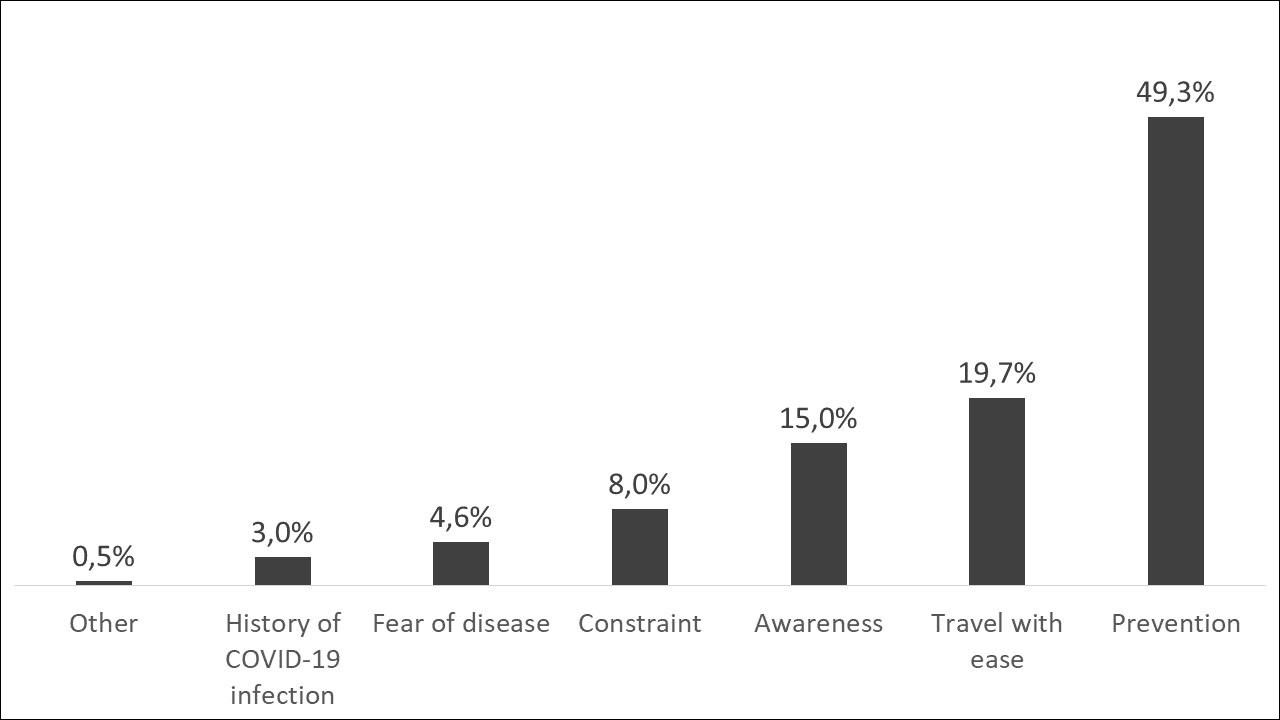

Supplement: Supplementary file 5 — Supplementary Material 5 [file 40794_2024_240_MOESM5_ESM.tif]

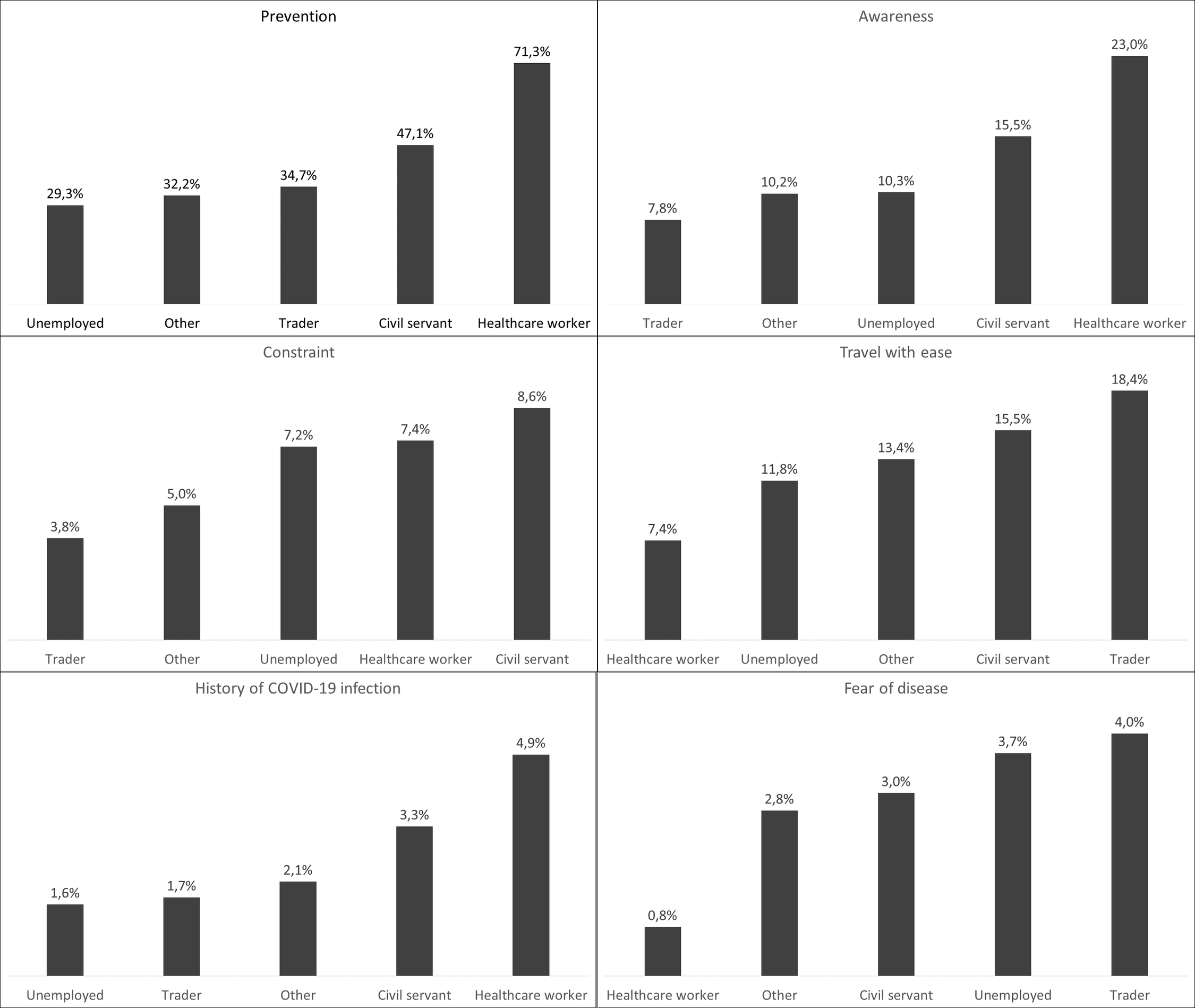

Supplement: Supplementary file 6 — Supplementary Material 6 [file 40794_2024_240_MOESM6_ESM.tif]

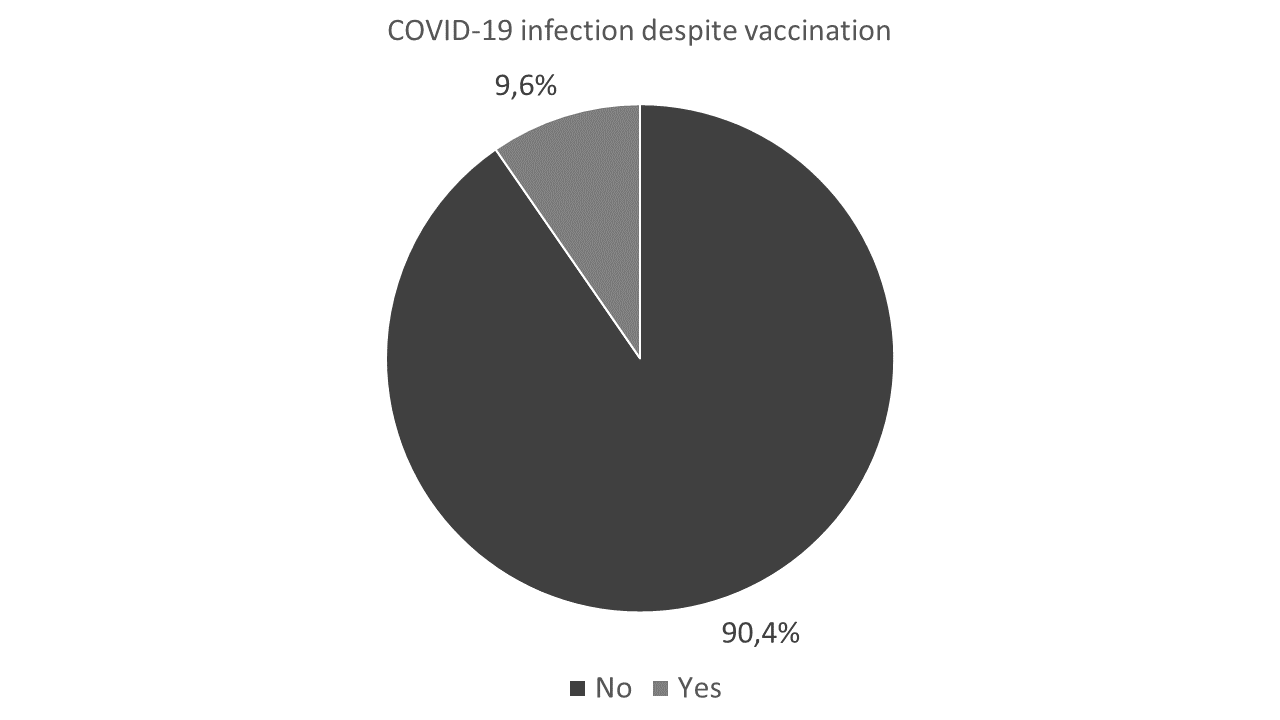

Supplement: Supplementary file 7 — Supplementary Material 7 [file 40794_2024_240_MOESM7_ESM.tif]

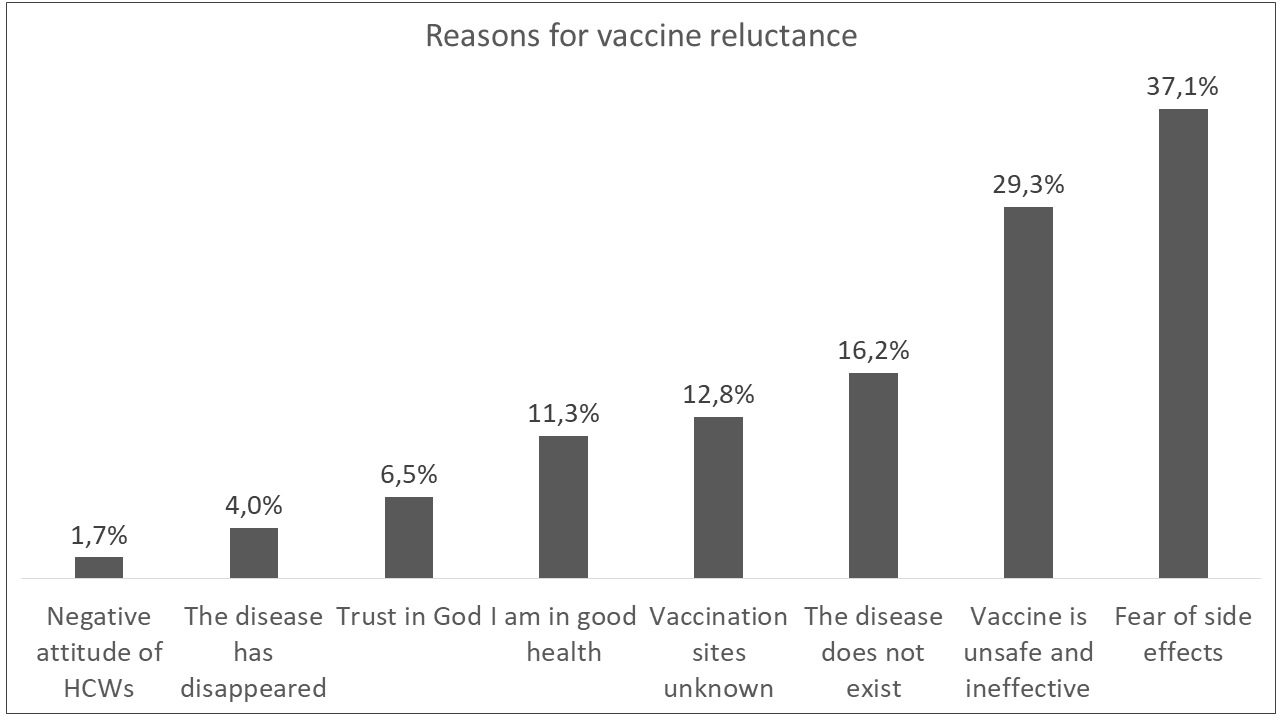

Supplement: Supplementary file 8 — Supplementary Material 8 [file 40794_2024_240_MOESM8_ESM.tif]

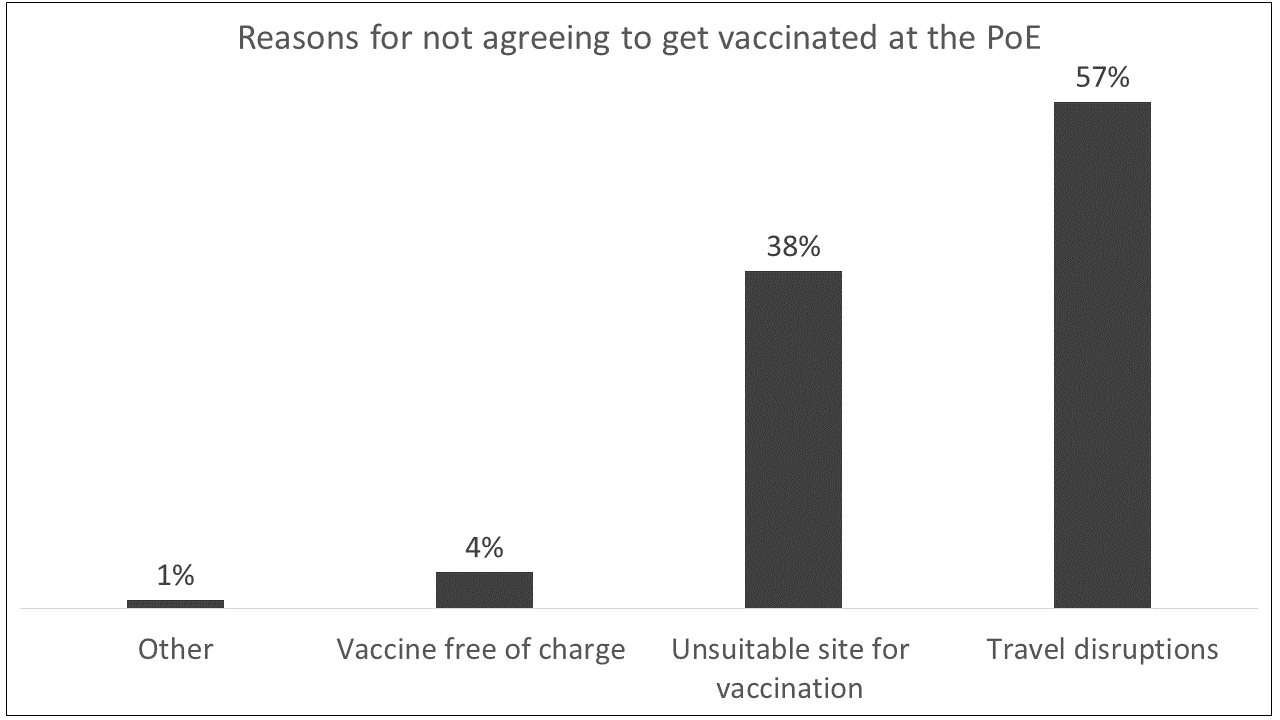

Supplement: Supplementary file 9 — Supplementary Material 9 [file 40794_2024_240_MOESM9_ESM.tif]
